# ERβ promotes Aβ degradation via the modulation of autophagy

**DOI:** 10.1038/s41419-019-1786-8

**Published:** 2019-07-22

**Authors:** Yong Wei, Jiawei Zhou, Jun Wu, Jian Huang

**Affiliations:** Hubei Key Laboratory of Cell Homeostasis, College of Life Sciences, Wuhan University, Wuhan, Hubei PR China

**Keywords:** Macroautophagy, Cellular neuroscience

## Abstract

Alzheimer’s Disease (AD) is the most common neurodegenerative disorder in the elderly. Beta-amyloid (Aβ) peptide accumulation is considered as a primary cause of AD pathogenesis, with defective autophagy in patients’ brains. Enhanced autophagic activity has been reported to promote Aβ clearance in vitro and in vivo models. Meanwhile, there is growing evidence that estrogen receptor β (ERβ) is a viable therapeutic target that can ameliorate the pathological features associated with AD. Very little is known about the detailed molecular mechanisms underlying the relationship between ERβ, autophagy, and Aβ degradation in AD. This study aims to uncover whether ERβ participates in autophagy and promotes extracellular Aβ_1–42_ degradation through the autophagy–lysosome system. Here we find that overexpression of ERβ caused autophagic activation as seen by increased microtubule-associated protein 1 light chain 3-II (LC3-II), SQSTM1 (sequestosome 1) degradation, LC3 punctate distribution, autophagosome, and autolysosome accumulation. In addition, we show that ERβ could induce autophagy through direct protein–protein interaction with ATG7 (E1-like enzyme). Furthermore, ERβ-mediated decrease in Aβ_1–42_ was blocked by the autophagy inhibitor chloroquine (CQ) in SH-SY5Y cells and the HEK293T (AβPPsw) model. Aβ_1–42_ or CQ induced cytotoxicity was restored by a selective ERβ activator diarylpropionitrile (DPN). Collectively, these data indicate that overexpression of ERβ exerts a neuroprotective effect through interacting with ATG7 protein and further enhances autophagy–lysosomal activity for Aβ_1–42_ clearance at the cellular level.

## Introduction

Alzheimer’s disease (AD) is a progressive neurodegenerative disease and is a primary cause of age-related disability and death in the world^1^. It is characterized by severe memory loss, cognitive impairment, and behavior changes. At the pathological level, extracellular deposition of plaques and intracellular neurofibrillary tangles are held to be the two major hallmarks of AD patients’ brains. Aβ_1–40_ and Aβ_1–42_, the main components of amyloid plaque, are products of amyloid precursor protein (APP), which is cleaved by β-secretase and γ-secretase complex. Aβ_1–40_ is the most abundant specie under physiological conditions^2^. In AD brain, Aβ_1–42_ is the most toxic specie due to its high hydrophobicity, resulting in a high tendency for aggregation^3^. Abnormally increased level of beta-amyloid (Aβ) is associated with the progression of AD, as it induces oxidative injury, neuroinflammation response, synaptic dysfunction, and neuron death^4^. Furthermore, studies show that 90–95% of all AD cases are sporadic, resulting from impaired clearance of Aβ^5^. Taken together, these data indicate that promoting Aβ clearance would be a potent therapeutic target for AD treatment.

Epidemiological studies have shown that women are more susceptible to AD than men, owing to the brain estrogen deficiency during menopause^6^. Ovariectomized-induced estrogen deficiency accelerates the Aβ plaque deposition in the AD mice model, while estrogen treatment reversed it^7,8^. Such evidence would help elucidate the neuroprotective actions of estrogen against Aβ. The physiological functions of estrogen are mainly regulated by estrogen receptor α (ERα) and estrogen receptor β (ERβ). Both receptors have been reported to decrease with age in the brain of rats and mice^9,10^. Unlike ERα, which has high distribution mainly in reproductive organs, ERβ has a prominent role in the nervous system^11,12^. Considering the side effect of ERα activation on reproductive organs under estrogen therapy^13^, selective activation of ERβ has been regarded as a potential valid target for AD therapy. Accumulating evidence shows that estrogen could mediate ERβ activation to stimulate the degradation of Aβ by upregulating Aβ-degrading enzymes in vivo and in vitro^14,15^. In addition, compared with the age-matched controls, the AD group shows reduced expression of ERβ in whole cell lysates along with more Aβ deposition^16^. Therefore, these data provide evidence that ERβ activation will benefit Aβ degradation in AD.

Macroautophagy (hereafter referred to as autophagy) is a necessary cellular process of lysosomal degradation that turns over intracellular cytoplasmic proteins and organelles, which helps maintain cellular homeostasis and neuronal health. During autophagy, small membrane structures called phagophore grows and gradually encloses cellular cargo, forming autophagosomes. Then, the autophagosomes fuse with lysosomes, forming autolysosomes and contribute to the recycling of autophagosomal components^17^. Enhanced autophagy flux and lysosomal activity promote Aβ to be engulfed by autophagic vacuoles (AVs), which then fuse with the lysosome and are recycled^18–21^. However, deficits in the autophagy–lysosome pathway exert an important role in the pathogenesis of AD, including the increased distribution of Aβ and the dysfunction of Aβ degradation^22^. Autophagy–lysosome is believed to be another major Aβ clearance route in addition to the different Aβ-degrading enzymes^23^. These results suggest that modulating the autophagy–lysosomal pathway can be a promising therapy for Aβ degradation in AD.

Based on the evidence cited above, we hypothesized that activation of autophagy mediated through ERβ overexpression can be associated with Aβ degradation. Although several reports demonstrated that ERβ could induce autophagy in various types of human cancers^24,25^, there is no direct evidence of a relationship between ERβ and autophagy. In addition, whether ERβ can activate autophagy in the nervous system remains unclear. Our data provide evidence for a role of ERβ in autophagy and the C/D region of ERβ plays a dominant role in the interaction between ERβ and ATG7, which has been shown to decrease in the AD mice model^26^. In addition, we observed that ERβ-induced Aβ degradation was significantly blocked by autophagy inhibition. We further found that Aβ_1–42_ or chloroquine (CQ) induced cytotoxicity could be suppressed by an ERβ agonist diarylpropionitrile (DPN). To the best of our knowledge, our data demonstrate for the first time that the degradation of extracellular Aβ fibrils by ERβ is dependent on the autophagic process.

## Materials and methods

### Cell culture and reagents

The human neuroblastoma SH-SY5Y cell line is thrice-cloned originally from SK-N-SH and widely used as an in vitro model for neuroscience research^27^. SH-SY5Y, HEK293T, and mouse embryonic fibroblasts (MEFs) were cultured in Dulbecco’s modified Eagle’s medium (Gibco) supplemented with 10% (v/v) Fetal Bovine Serum (Biological Industries) and 1% penicillin/streptomycin (Gibco, USA). HEK293T cells were transfected with a Swedish mutant AβPP695 (AβPPsw) or empty vector labeled with GFP tag. Stably transduced cells were selected for neomycin resistance using G418 (Sigma, USA). All cells were routinely cultured in a humidified atmosphere of 5% CO_2_ at 37 °C incubator. Reagents used in this study were DPN (HY-12452; an ERβ-selective agonist) and CQ (HY-17589; a lysosome inhibitor) from MedChemExpress (NJ, USA). All chemical reagents were dissolved in DMSO and the final DMSO concentrations in each experiment were <0.2%.

### RNA interference

*ERβ* silencing siRNA, *ATG7* silencing siRNA, and control siRNA were purchased from GenePharma (Suzhou, China). The sequences of *ERβ*, *ATG7*, and control siRNA were as follows: ERβ sense, 5′-CCAGCCAUGACAUUCUAUATT-3′ and antisense, 5′-UAUAGAAUGUCAUGGCUGGTT-3′; *ATG7*#1 sense, 5′-GGUCAAAGGACG-AAGAUAATT-3′ and antisense, 5′-UUAUCUUCGUCCUUUGACCTT-3′; *ATG7*#2 sense, 5′-GCCUCUCUAUGAGUUUGAATT-3′ and antisense, 5′-UUCAAACUCA-UAGAGAGGCTT-3; control siRNA sense, 5′-UUCUCCGAACGUGUCACGUTT-3′ and antisense, 5′-ACGUGACACGUUCGGAGAATT-3′. These kinds of siRNA oligonucleotides were transfected into SH-SY5Y cells at 100 nM using Lipofectamine 2000 (Invitrogen, USA) according to the manufacturer’s instructions.

### Plasmids and deletion mutants construction

EGFP-C1-ERβ was a gift from Michael Mancini (Addgene plasmid #28237); EGFP-C1-ATG7 was kindly provided by Rongjia Zhou (Wuhan University, Wuhan, China). ERβ and its deletion mutants were cloned into the pHAGE-puro plasmid with a Flag tag. The EGFP-C1-ERβ plasmid was used as the template. The resulting plasmids were named pHAGE-puro-ERβ, ERβ-ΔD/F, ERβ-C/D, ERβ-ΔC/F, and ERβ-ΔA/D, respectively. To construct the pGL3-promoter-ATG7 luciferase reporter plasmid, a fragment ranging from −2015 to +112 bp of the human ATG7 promoter (GenBank accession number, NC_000003.12) was PCR amplified from HEK293T genomic DNA and inserted into MluI and Xhol sites of a luciferase reporter vector (pGL3-promoter). The primer sequences are as follows: sense, 5′-GCGACGCGTAAGGTCAAACACAGTCCTTCT-3′ and antisense,5′-CCGCTCGAGCTTACCGCCGCTCAACTT-3′. All constructs were confirmed by DNA sequence analysis. Plasmids were transfected into SH-SY5Y or HEK293T using Lipofectamine 2000 for 48 h according to the manufacturer’s protocols.

### Western blot and antibodies

Cortex lysates from wild type and 2xTg-AD (APPswe/PSENldE9) mice were kindly provided by Dr Vilhelm A. Bohr (National Institutes of Health). Cellular protein was extracted with RIPA buffer (Beyotime, Shanghai, China) supplemented with complete protease inhibitor mixture (Roche, Mannheim). Subsequently, the western blot assay was performed by following the previous descriptions^28^. The antibodies used include: LC3B (L7543), Flag (F7425), and β-actin (A1978) from Sigma-Aldrich (St. Louis, MO, USA); ATG5 (#2630) and ATG7 (#8558) from Cell Signaling technology (San Diego, USA), p62/SQSTM1 (18420-1-AP), GFP (50430-2-AP), cathepsin D (CTSD) (21327-1-AP), and ERβ (14007-1-AP) from Proteintech (Wuhan, China); Lysosome-associated membrane protein type 2 (LAMP2) (A14017) and BACE1 (A5266) from Abclonal (Wuhan, China); α7nAChR (501588) from ZENBIO (Chengdu, China); peroxidase-conjugated immunopure goat anti-rabbit and anti-mouse IgG (HL) from Abclonal. Enhanced chemiluminescence horseradish peroxidase was used to visualize protein bands. NIH ImageJ software was used to measure the intensity of the bands.

### Immunoprecipitation

SH-SY5Y cells were harvested and lysed in immunoprecipitation buffer (50 mM Tris-HCl, pH8.0, 150 nM Nacl, 2 mM EDTA, 10% glycerol, 1% Triton X-100) supplemented with protease inhibitors. Lysates were transferred to 1.5 ml tubes, rotated for 1 h at 4 °C, and centrifugated at 13,000 rpm for 25 min. The supernatant was then incubated with control IgG (Santa Cruz Biotechnology, sc-2027 and sc-2025) or antibodies against ERβ (Santa Cruz Biotechnology; sc-373853) or ATG7. Next, protein A+G Agarose beads (20 μ, Beyotime, P2012) were added into the immunoprecipitation reaction with an additional 4 h of rotation at 4 °C. The antigen–antibody complexes were precipitated by a quick centrifugation and washed four times with immunoprecipitation buffer. After a quick centrifugation, the sediment was resuspended in SDS loading buffer. The mixture was then boiled for 10 min before immunoblot analysis.

### RNA isolation and quantitative real-time PCR (qRT-PCR)

Total RNA was extracted using Trizol extraction kits (Invitrogen, US) according to manufacturer’s protocol and then treated with RNase-free DNase I (TaKaRa, Japan) to avoid potential DNA contamination. Reverse transcription was performed using the M-MLV Reverse Transcriptase (Promega, #M1701) and random primers. Approximately 1 μg of the total RNA was used as the template for reverse transcription. The cDNA was diluted to 100 ng/μL for use in qRT-PCR. The following primers were used: *GAPDH* sense, 5′-TGCACCACCAACTGCTTAGC-3′ and antisense, 5′-GGCATGGACTGTGGTCATGAG-3′; *ATG5* sense, 5′-TTCAATCAGGTTTGGTGGAGGC-3′, and antisense, 5′-ATGGCAGTGGAGGAAAGCAGA G-3′, *ATG7* sense, 5′-TGCTATCCTGCCCTCTGTCTT-3′ and antisense, 5′-TGCCTCCTTTCTGGTTCTTTT-3′; ERβ sense, 5′-TCCATGCGCCTGGCTAAC-3′ and antisense, 5′-CAGATGTTCCATGCCCTTGTTA-3′. Data analysis was performed using Bio-rad CFX manager system, using GAPDH as a reference transcript.

### Transmission electron microscopy (TEM)

SH-SY5Y cells were washed and fixed at room temperature (RT) for 1 h in 2.5% glutaraldehyde supplemented with 0.1 M phosphate buffer saline (PBS), and then postfixed in 1.0% osmium tetroxide for 3 h. Next, cells were scraped, spun down, serially dehydrated in ethanol baths, and embedded in blocks of epon Araldite. Ultrathin sections (60–80 nm) were made using an Ultracut Microtome (UC7; Leica), stained with 4% aqueous uranyl acetate and lead citrate for 5 min, and then performed using a TEM (Tecnai G2 20 Twin, FEI) at 200 KV.

### Enzyme-linked immunosorbent assay (ELISA)

Human Aβ_1–42_ was purchased from Qiangyao Biotechnology (Shanghai, China). Aβ_1–42_ fibrillar oligomers were prepared by initially dissolving the lyophilized peptide in NaOH as the previous description^29^. After treatment, the medium was collected and Aβ_1–42_ levels were measured by ELISA kits (CUSABIO BIOTECH, China) according to the manufacturer’s protocols.

### Luciferase reporter assay

For dual luciferase analysis, ~1 × 10^5^ HEK293T cells were seeded on 24-well plates. Each well was transfected with 400 ng luciferase reporter plasmid and 2 ng of internal control plasmid pRL-CMV vector (Promega, USA) using Lipofectamine 2000. Twenty-four hours later, cells were transfected with pHAGE-puro or pHAGE-puro-ERβ plasmid for another 24 h. Next, cells were harvested and lysed with passive lysis buffer (Promega) and Luciferase units were measured by the dual luciferase assay system protocol (Promega).

### LysoTracker labeling

SH-SY5Y cells (~1 × 10^5^) were transferred to six-well plates. After treatment with DPN (10 nM) for 24 h, cells were incubated with 100 nM Lyso-Tracker Red (Molecular probes, Invitrogen, OR, USA) for 30 min at 37 °C in a humidified atmosphere of 5% CO_2_. Cells were then washed twice in PBS and immediately visualized in the culture medium directly under confocal microscopy.

### Immunofluorescence staining

Cells were grown on coverslips and fixed in PBS containing 4% paraformaldehyde for 20 min at RT. After washing twice with PBS, the cells were permeabilized with 0.1% Triton X-100 and blocked in 1% Albumin Bovine V for 30 min at RT. The coverslips were then incubated with primary antibodies: light chain 3 (LC3) antibody (rabbit), ERβ (mouse, SAB2702146, Sigma), and ATG7 (rabbit) diluted in PBS (0.01% Triton X-100) overnight at 4 °C. After three washes in PBS, secondary antibodies were applied. Alexa Fluor 594 conjugated anti-rabbit IgG (Red) was used for LC3 and ATG7. Alexa Fluor 488 conjugated anti-mouse IgG (Green) was used for ERβ. Nuclei were counterstained by 4′-6-diamidino-2-phenylindole (DAPI) (Beyotime, C1005). Cell images were captured with a confocal microscopy (Leica TCS SP8, Germany).

### Assessment of cell viability

The MTT (3-(4, 5-dimethylthiazol-2-yl)-2, 5-diphenyltetrazolium bromide) cell viability assay was employed to measure the protective effect of DPN in Aβ_1–42_ or CQ damaged SH-SY5Y cells. After various treatments, cell viability was then assayed with the MTT method.

### Statistical analyses

All assays were repeated at least three times and continuous variables were expressed as mean ± SD. All data analyses were performed using GraphPad Prism curve comparisons. Differences were considered statistically if *p* < 0.05. No statistical methods were used to predetermine sample size.

## Results

### ERβ activates autophagy in SH-SY5Y cells

**Fig. 1:**
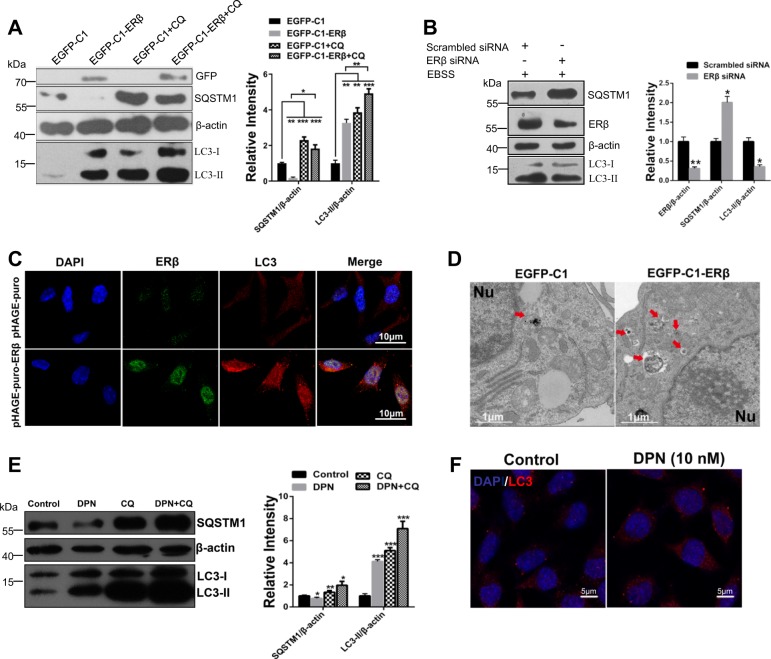
ERβ protein induces autophagy in SH-SY5Y cells. **a** EGFP-C1 and EGFP-C1-ERβ groups were treated with chloroquine (10 µM) for 12 h or not. The levels of ERβ, SQSTM1, and LC3-II were compared with western blotting using related antibodies. β-actin was used as an equal loading control in this text. Densitometry analysis of LC3-II and SQSTM1 levels relative to β-actin is also shown (right). **b** After EBSS treatment for 1 h, cells were transfected with scrambled siRNA or *ERβ* siRNA for 48 h, and then cell lysates were analyzed by related antibodies. Bar graph (right) indicates the relative ratio of LC3-II, SQSTM1, and ERβ to β-actin in SH-SY5Y. **c** Cells were transfected with pHAGE-puro and pHAGE-puro-ERβ plasmids, the cytoplasm distribution of endogenous LC3 punctate structures was observed by confocal microscopy in SH-SY5Y cells. DNA was stained by DAPI. Scale bar = 10 μm. **d** Cells were transfected with EGFP-C1 and EGFP-C1-ERβ plasmids, representative electron micrographs were used to monitor autophagic vacuoles. Scale bar = 1 μm. **e** Cells were treated with DPN (10 nM), CQ (10 μM), and DPN plus CQ for 12 h. Cell lysates were analyzed by immunoblotting for LC3-II, SQSTM1, and β-actin protein expression. Bar graph (right) indicates the relative ratio of LC3-II and SQSTM1 to β-actin in SH-SY5Y. **f** Cells were treated with DPN (10 nM), the cytoplasm distribution of endogenous LC3 punctate structures was observed by confocal microscopy in SH-SY5Y cells. Scale bar = 5 μm. Data shown are mean ± S.D. of three independent experiments. (*P < 0.05; **P < 0.01; ***P < 0.001)

To investigate whether ERβ could induce autophagy, we quantified the level of autophagy through various methods. During the autophagy–lysosome process, the unlipidated cytosolic form LC3-I is converted to lipidated form LC3-II and SQSTM1 protein is continuously degraded by autolysosomes^30,31^. As shown in Fig. 1a, overexpression of ERβ contributed to enhanced autophagic flux as evidenced by the increased LC3-II expression and degradation of SQSTM1 protein. However, the EGFP-C1-ERβ group, as compared with the EGFP-C1 group, shown more LC3-II accumulation upon CQ treatment. CQ is often used to raising the intralysosomal pH, blocking the fusion of autophagosomes with lysosomes, as demonstrated by a marked accumulation of LC3-II^32^. ERβ-induced SQSTM1 decrease was also blocked in the presence of CQ. The transfection efficiency was determined by immunofluorescence microscopy in SH-SY5Y cells (Fig. S1B). Next, knockdown of ERβ reduced LC3-II and increased SQSTM1 followed by the treatment of Earle’s Balanced Salt Solution (EBSS) (starvation condition) for 1 h (Fig. S1C and Fig. 1b). Such evidence suggests that ERβ is positively related to autophagy status in SH-SY5Y cells. To confirm the effect of ERβ on autophagy activation, we also observed the cytoplasm distribution of endogenous LC3 punctate structures by confocal microscopy. As shown in Fig. 1c, ERβ transfection increased the LC3-positive subcellular structures. Consistent with the above results, TEM analysis revealed that there were abundant autophagosomes and autolysosomes in the ERβ transfected group (Fig. 1d). Collectively, these data suggest that ERβ activates the formation of autophagosome in SH-SY5Y cells. To further confirm the role of ERβ in autophagy, an ERβ-selective agonist DPN was used to measure the autophagy level. As shown in Fig. S1D, the level of LC3-II was significantly increased and the expression of SQSTM1 was decreased in a concentration-dependent manner. Besides, DPN (10 nM)-induced LC3-II increase was potentiated under CQ treatment, consistent with an increased upstream autophagosome formation (Fig. 1e). DPN (10 nM) also increased the cytoplasm distribution of endogenous LC3 punctate structures (Fig. 1f). Altogether, our results demonstrate that ERβ contributes to autophagy activation and the fusion of autophagosomes and lysosomes.

### ERβ induces autophagy through ATG7 protein

**Fig. 2:**
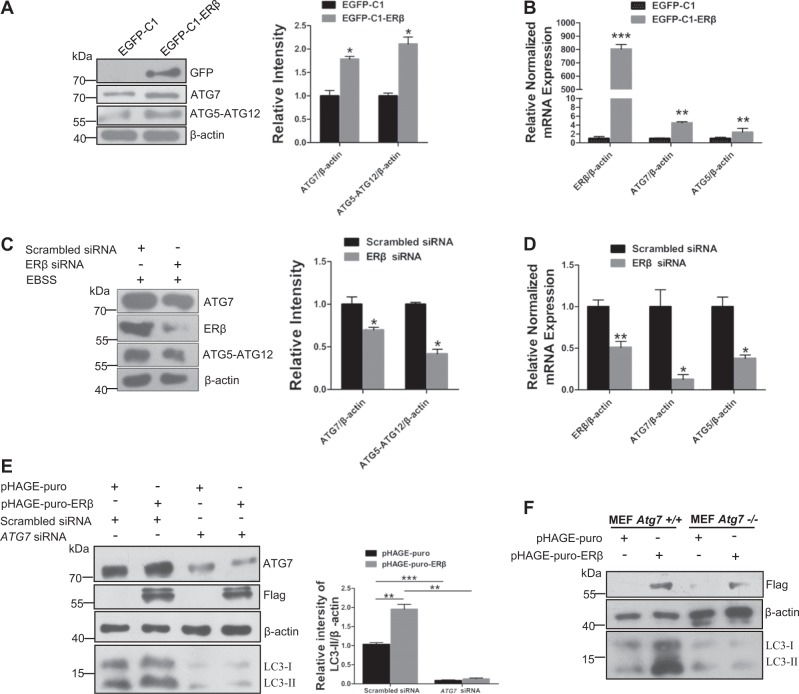
ERβ regulates ATG7 expression in SH-SY5Y cells. **a** Cells were treated as described in (Fig. 1d), protein contents of ATG7, ERβ, and ATG12–ATG5 conjugation were examined by immunoblot in SH-SY5Y cells. Bar graph indicates the relative ratio of ATG7, ATG5-ATG12 to β-actin in SH-SY5Y cells (right). **b** qRT-PCR was performed to measure the transactivity of ERβ, ATG7, and ATG5 in SH-SY5Y cells followed by transient transfection of EGFP-C1 and EGFP-C1-ERβ plasmids for 24 h. **c** Cells were treated as described in (Fig. 1b), immunoblot analysis was performed for ATG7, ERβ, and ATG5-ATG12 complex (left). Right panel, quantification of ATG7, ERβ, and ATG5-ATG12 complex expression using ImageJ software. **d** mRNA levels of ERβ, ATG7, and ATG5 in SH-SY5Y cells treated with scrambled siRNA or *ERβ* siRNA for 24 h. **e** SH-SY5Y cells were transiently transfected with pHAGE-puro or pHAGE-puro-ERβ plasmid. After 12 h transfection, cells were retransfected with *ATG7* siRNA and a nonspecific RNAi control for 36 h. ATG7, ERβ, and LC3-II levels were tested by western blot (left). Right panel, quantification of LC3-II expression using ImageJ software. **f** pHAGE-puro or pHAGE-puro-ERβ plasmid was transfected into MEF *Atg7*^*+/+*^ and *MEF Atg7*^−*/*−^ cells. After 48 h culture, cells were harvested for LC3 detection. Data shown are mean ± S.D. of three independent experiments. (*P < 0.05; **P < 0.01; ***P < 0.001; ****P < 0.0001)

Given that an interaction between ERβ and ATG7 was predicted by the autophagy regulatory network database^33^, we further explored whether ATG7 is involved in ERβ-regulated autophagy in SH-SY5Y cells. To assess the possibility that ATG7 participates in ERβ-induced autophagy, we tested the levels of ATG7 and ATG5-ATG12 complex in SH-SY5Y cells. The ATG7-regulated catalysis of ATG12 conjugation to ATG5 is an essential step for the formation of autophagosomal structures^34^. Remarkably, we observed that ERβ overexpression increased the ATG7 expression and formation of the ATG5-ATG12 complex in SH-SY5Y (Fig. 2a), while ERβ silence had an opposite effect (Fig. 2c), suggesting an important role of ERβ in ATG7 expression. Meanwhile, qRT-PCR performance showed that ERβ could also affect the ATG7 and ATG5 at the transcriptional level (Fig. 2b, d). Next, we explored whether the effect of ERβ on autophagy is dependent on ATG7. We designed two siRNAs of *ATG7*, and the siRNA efficiency was tested by western blot (Fig. S2A). As anticipated, overexpression of ERβ-induced LC3-II accumulation was impaired in the presence of *ATG7* siRNA (Fig. 2e). Similarly, compared with MEF *Atg7*^*+/+*^ cells, ERβ-induced LC3-II expression was blocked in MEF *Atg7*^*−/−*^ cells (Fig. 2f). The expression of ATG7 in both wide-type and knockout MEF cells was tested by immunofluorescence and western blot (Fig. S2B, C). To further confirm that ERβ affects autophagy through actions on ATG7, we tested the upstream marker of autophagy. ULK1 (unc-51 like autophagy activating kinase1) is an important regulator in the autophagy pathway, and the phosphorylation of ULK1 at Ser757 is closely related to the repression of autophagy induction^35^. Our results showed that ERβ could not affect phospho-ULK1level under DPN treatment (Fig. S2D). Thus, these results suggest that ATG7 is a prerequisite for ERβ-regulated autophagy in SH-SY5Y cells.

### ERβ interacts and colocalizes with ATG7

**Fig. 3:**
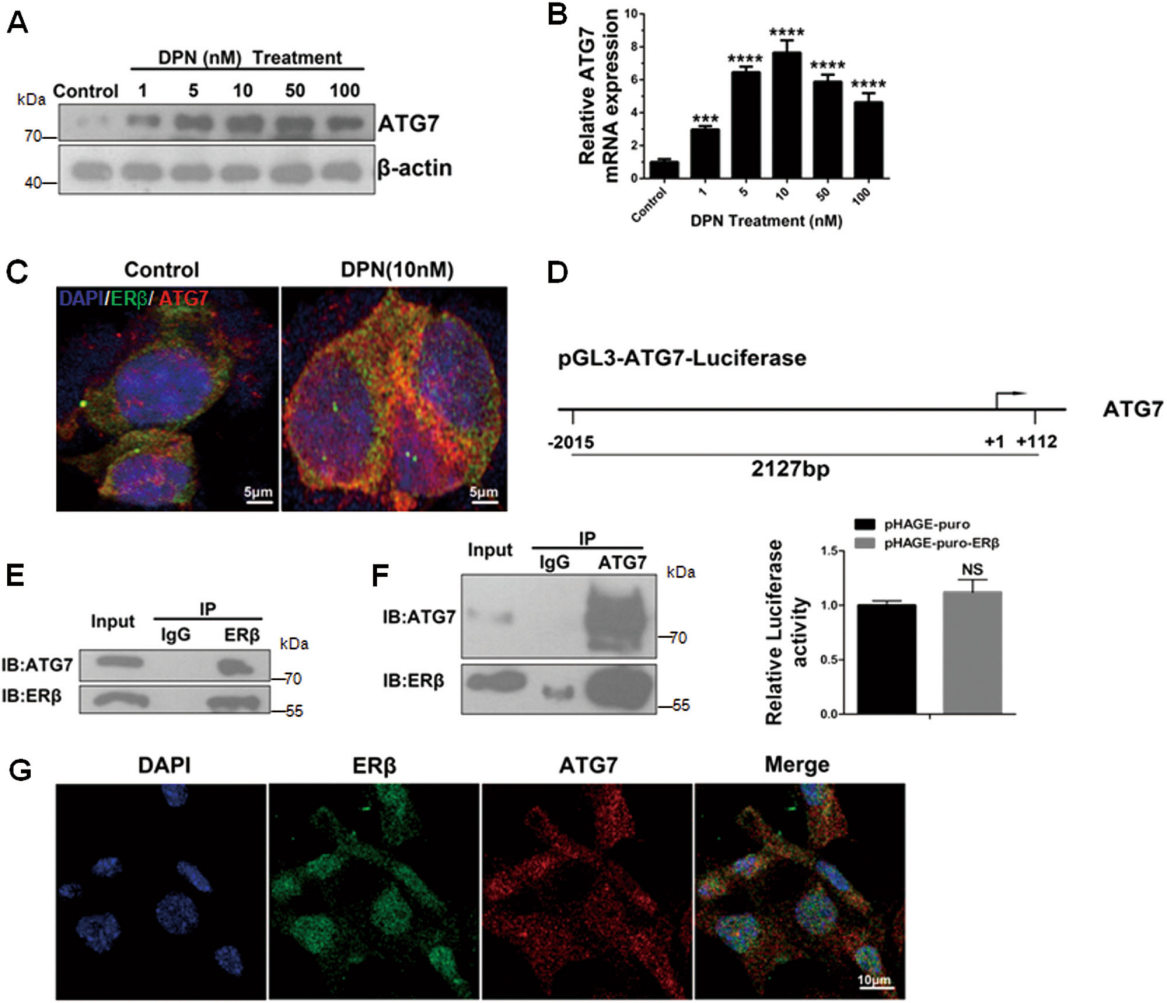
ERβ interacts and colocalizes with ATG7. **a** Cells were treated as described in (Fig. S1D), the ATG7 protein expression was detected by western blot. **b** Cells were treated as described in (Fig. S1D), Quantitative PCR was performed to measure the transactivity of ATG7 in SH-SY5Y cells. **c** The Confocal assay was performed to measure the colocalization between ERβ and ATG7 following the treatment of DPN for 24 h. **d** Schematic representation of the ATG7 promoter region (−2015 to +112 bp) (top). To detect the effect of ERβ on ATG7 promoter activity, HEK293T cells were transiently cotransfected with pHAGE-puro or pHAGE-puro-ERβ plasmid and ATG7 luciferase plasmid for 24 h (bottom). **e, f** SH-SY5Y whole cell lysates collected from 10 cm dishes were subjected to immunoprecipitation with an anti-ERβ or anti-ATG7 antibody. An IgG antibody was used as a control. **g** SH-SY5Y cells were immunostained for endogenous ERβ and ATG7 to observe their colocalization. Colocalization appears yellow. Pearson’s index (0.44) of colocalization of ERβ with ATG7. Scale bar = 10 μm. Data shown are mean ± S.D. of three independent experiments. (*P < 0.05; **P < 0.01; ***P < 0.001)

Since ERβ overexpression could regulate ATG7, we investigated the expression of ATG7 in response to increasing concentrations of DPN. As shown in Fig. 3a, ATG7 protein was positively regulated by DPN in a dose-dependent manner from 1 to 50 nM. Similar results were obtained from ATG7 mRNA expression analysis, with a peak at 10 nM (Fig. 3b). Here, we also observed that the endogenous colocalization of both proteins was increased under the treatment of DPN (10 nM) for 24 h (Fig. 3c). Such a result prompts us to speculate whether the mechanism of ERβ-regulated autophagy is due to the binding of ATG7 promoter directly. To assess this hypothesis, dual luciferase reporter analysis was performed in HEK293T cells. However, there was no change in the activity of luciferase in cells overexpressing ERβ plasmid (Fig. 3d), suggesting that another mechanism exists between ERβ and ATG7. In our present study, endogenous ERβ and endogenous ATG7 were reciprocally immunoprecipitated in SH-SY5Y cells (Fig. 3e, f). The Confocal microscopy assay also revealed the colocalization of ERβ with ATG7 mainly in the cytoplasm of SH-SY5Y (Fig. 3g). Collectively, these results demonstrate that ERβ activates autophagy via the binding of ATG7 protein rather than the ATG7 promoter.

### C/D domain of ERβ plays a significant role in ERβ/ATG7 interaction

**Fig. 4:**
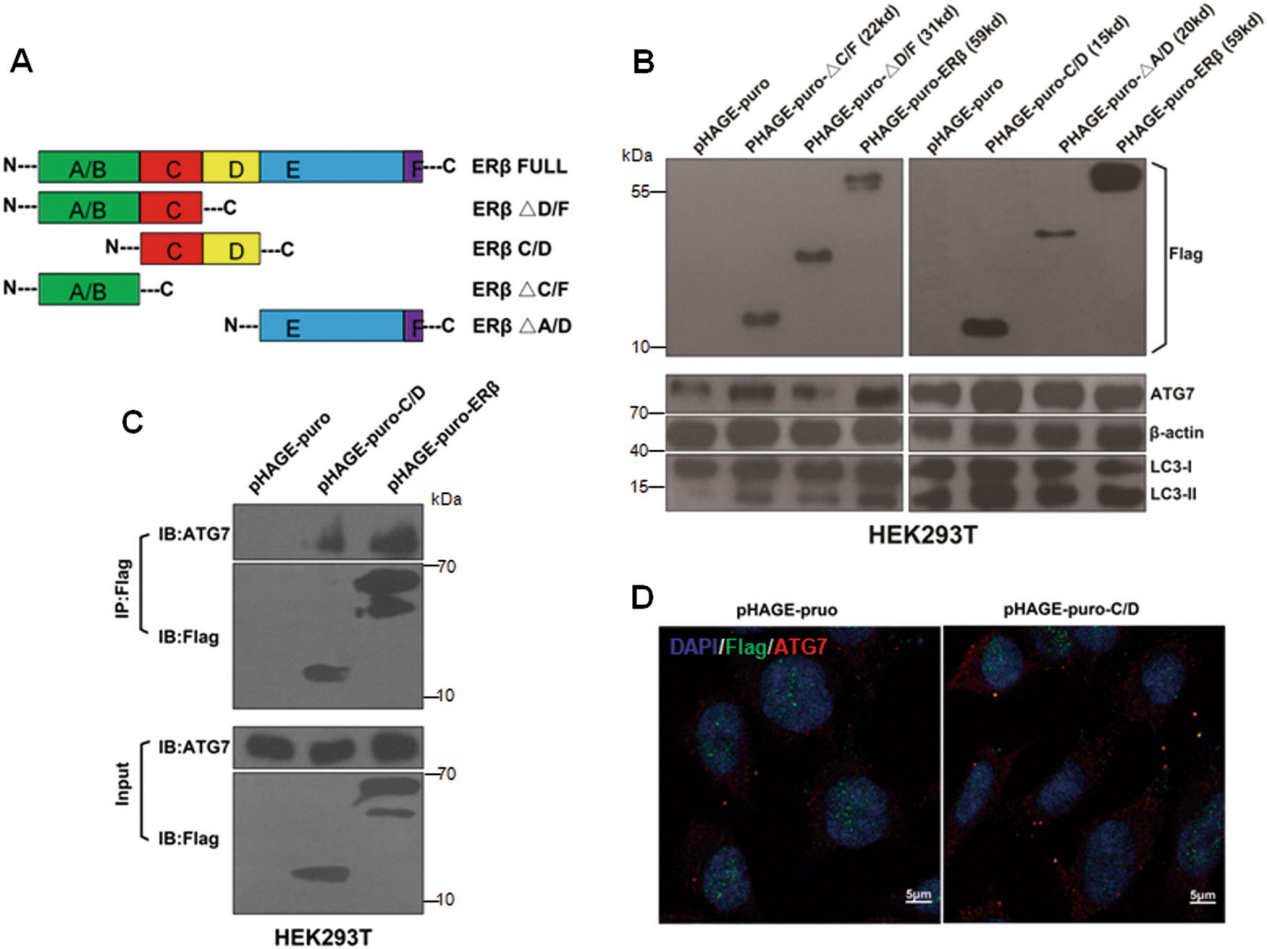
C/D domain of ERβ contributes to autophagy. **a** Schematic diagram of various ERβ deletion mutants. **b** HEK293T cells were transfected with ERβ, ERβ deletion mutants, or vector control Flag-tagged plasmids. After 48 h treatment, cells were examined for Flag, ATG7, and LC3-II by western blot. **c** HEK293T cells were transfected with a plasmid encoding Flag-C/D and either Flag-ERβ or empty vector. The cell lysates were immunoprecipitated with Flag antibody and blotted with ATG7 and Flag antibodies. **d** SH-SY5Y cells were transfected with ERβ-C/D and its control plasmid for 48 h. Then, cells were immunostained for endogenous ERβ and exogenous Flag to observe their colocalization under confocal microscopy. Scale bar = 5 μm. Data shown are mean ± S.D. of three independent experiments. (*P < 0.05; **P < 0.01; ***P < 0.001)

ERβ is a nuclear transcription factor, with five distinct functional domains (A–F)^36^. To identify which domain of ERβ is responsible for the binding to ATG7 protein, a series of Flag-tagged deletion mutants of ERβ, named ERβ-ΔD/F, ERβ-C/D, ERβ-ΔC/F, and ERβ-ΔA/D were constructed (Fig. 4a). HEK293T cells overexpressing ERβ, ERβ deletion mutants or vector control were examined for ATG7 and LC3-II. ERβ-C/D plasmid transfection, as well as ERβ, had a remarkable increase in ATG7 and LC3-II expression compared with the control group, while other mutants showed a light increase or stable level (Fig. 4b), suggesting the main requirement of C/D region in ERβ-mediated autophagy. In order to figure out if C/D domain could interact with ATG7, we performed immunoprecipitation experiment and found that ERβ could bind to ATG7 through C/D domain (Fig. 4c). To further explore the relationship between C/D domain of ERβ and ATG7, the confocal assay showed a cytoplasmic colocalization between Flag-tagged C/D domain and ATG7 in SH-SY5Y cells (Fig. 4d). Collectively, these data indicate that C/D domain of ERβ plays a significant role in the ATG7-dependent autophagy.

### ERβ overexpression enhances lysosomal function in SH-SY5Y cells

**Fig. 5:**
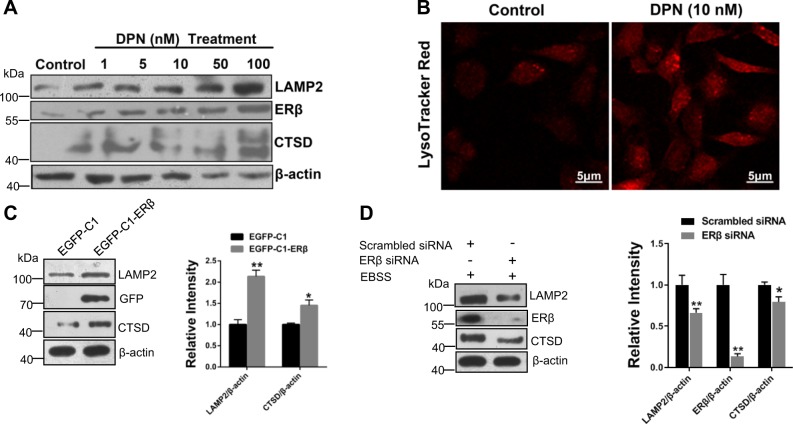
ERβ activation enhances the lysosome function in SH-SY5Y cells. **a** Cells were treated as described in (Fig. S1D), the LAMP2 and CTSD protein expressions were detected by western blot. **b** The fluorescence intensity of Lyso-Tracker Red was observed using confocal microscopy under the DPN (10 nM) treatment for 24 h. **c** Cells were treated as described in (Fig. 1d), then the LAMP2 and CTSD levels were tested by western blot (left). Right bar graph indicates the relative ratio of LAMP2 and CTSD to β-actin in SH-SY5Y cells. **d** Cells were treated as described in (Fig. 1b), then the LAMP2 and CTSD levels were tested by western blot (left). Right bar graph indicates the relative ratio of LAMP2 and CTSD to β-actin in SH-SY5Y cells. Data shown are mean ± S.D. of three independent experiments. (*P < 0.05; **P < 0.01; ***P < 0.001)

Since ERβ could enhance the autophagic flux from the above results, we would like to explore the lysosomal functions in SH-SY5Y cells further. We found that DPN could increase the expression of LAMP2 and CTSD in a dose-dependent manner from 1 to 100 nM in SH-SY5Y (Fig. 5a). To better understand the role of ERβ in the lysosome, we performed immunofluorescence labeling of lysosomes with LysoTracker-Red (Fig. 5b). The increased fluorescence intensity of Lyso-Tracker Red was observed using confocal microscopy, indicating the function of lysosome was reinforced. Besides, overexpression of ERβ increased the LAMP2 and CTSD level, while silencing ERβ reversed them in SH-SY5Y cells (Fig. 5c, d). These results indicate that ERβ overexpression enhances lysosomal function in SH-SY5Y cells.

### ERβ promotes extracellular Aβ_1–42_ degradation via autophagy–lysosome system

**Fig. 6:**
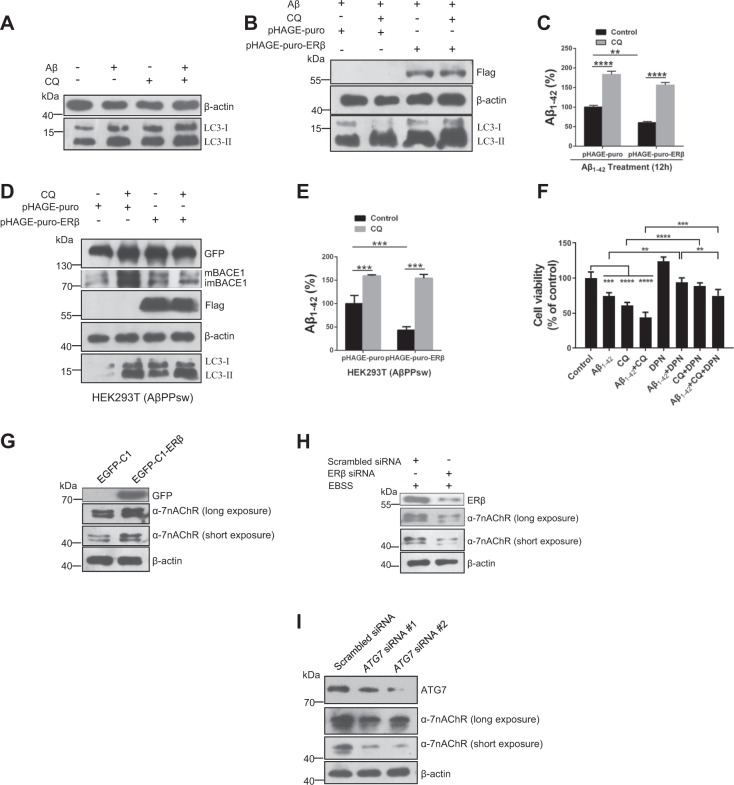
Enhanced autophagy promotes extracellular Aβ_1–42_ degradation in SH-SY5Y cells. **a** Cells were treated with Aβ (1 μM), CQ (10 μM), and Aβ plus CQ for 12 h. Cell lysates were analyzed by immunoblotting for LC3-II and β-actin protein expression. **b** After cells were transiently transfected with pHAGE-puro and pHAGE-puro-ERβ for 24 h, cells were treated with CQ 10 (μM) for 12 h. Next, cells were treated with Aβ_1–42_ Aβ fibrils for 12 h. LC3-II level was tested by western blot. **c** Cells were treated as described in **b**, the Aβ_1–42_ concentration was measured by an ELISA assay. **d** Cells were transfected with ERβ and vector plasmids under CQ treatment or not in the HEK293T (AβPPsw) model. Cell lysates were analyzed by immunoblotting for APP, BACE1, Flag, LC3-II, and β-actin protein expression. **e** Cells were treated as described in **d**, the Aβ_1–42_ concentration was measured by an ELISA assay. **f** Cells were treated with DPN (10 nM) or Aβ_1–42_ (5 μM) for 12 h and then added CQ (10 μM) for another 12 h. The MTT assay was used to test cell viability. **g** Cells were treated as described in (Fig. 1d), then the α7nAChR level was tested by western blot. **h** Cells were treated as described in (Fig. 1b), then the α7nAChR level was tested by western blot. **i** Cells were treated as described in (Fig. S2A), then the α7nAChR level was tested by western blot. Data shown are mean ± S.D. of three independent experiments. (*P < 0.05; **P < 0.01; ***P < 0.001; ****P < 0.0001)

Aβ_1–42_ has been reported as an autophagy inducer accompanied by increased LC3-II expression^37^. Our results also confirmed that Aβ_1–42_ (5 μM)-induced LC3-II protein increase could be enhanced by the cotreatment with CQ (10 μM) in SH-SY5Y cells (Fig. 6a). To value whether autophagy activation promotes extracellular Aβ_1–42_ degradation in SH-SY5Y, CQ was added to the medium of the pHAGE-puro and pHAGE-puro-ERβ group for 12 h. Next, cells were treated with Aβ_1–42_ fibrils for another 12 h. Afterwards, the inhibitory effect on autophagy–lysosome fusion was tested by western blot and the remaining Aβ_1–42_ concentration was measured by ELISA kits. As expected, CQ enhanced ERβ-induced LC3-II expression in the presence of Aβ_1–42_ compared with the control group (Fig. 6b). Further, the ELISA assay showed that overexpression of ERβ-induced extracellular Aβ_1–42_ degradation was blocked by CQ (Fig. 6c). On the other hand, we constructed a stable APP overexpression model in HEK293T cells (Fig. S3B–D). Our results showed that ERβ transfection increased LC3-II and had no effect on BACE1 expression in the HEK293T (AβPPsw) model (Fig. 6d). The ELISA assay also confirmed that ERβ could induce extracellular Aβ_1–42_ degradation via autophagy–lysosome pathway (Fig. 6e). To test whether ERβ-induced extracellular Aβ_1–42_ degradation has a neuroprotective role in SH-SY5Y cells. The MTT assay was performed to measure cell viability. Results certified that DPN could restore Aβ_1–42_ or CQ induced cytotoxicity in SH-SY5Y cells (Fig. 6f). These results support the finding that enhanced autophagic activity contributes to extracellular Aβ_1–42_ degradation and then exerts neuroprotective effects. However, it remains unclear how extracellular Aβ is taken up into the cytosolic compartment. Hung et al.^38^ show that LC3 overexpression reduces Aβ_1–42_ neurotoxicity through promoting alpha7 nicotinic acetylcholine receptor (α7nAChR) expression for extracellular Aβ_1–42_ binding and further activating autophagy for extracellular Aβ_1–42_ degradation in SH-SY5Y cells. Our results showed that ERβ overexpression enhanced α7nAChR expression, while ERβ siRNA reversed it (Fig. 6g, h). In addition, ATG7 knockdown reduced α7nAChR expression, suggesting that impaired autophagy may reduce α7nAChR expression (Fig. 6i). Taken together, the present results indicate that ERβ increases α7nAChR expression and enhances extracellular Aβ_1–42_ degradation via the autophagy–lysosome system in SH-SY5Y cells.

## Discussion

Our previous study showed that ERβ mediated-CyclinD1 degradation via autophagy plays an anti-proliferation role in colon cells. Here, for the first time, we demonstrated a novel function of ERβ in regulating autophagy-dependent extracellular Aβ_1–42_ degradation in SH-SY5Y cells. In particular, we found a direct link between the neuroprotection of ERβ and autophagy induction in SH-SY5Y cells. Overexpression of ERβ promoted the autophagosome formation and enhanced the lysosomal function in an ATG7-dependent manner, while knockdown of ERβ reversed it. Dissecting the mechanism of ERβ-regulated autophagy, we found that the C/D domain of ERβ plays a significant role in the ERβ–ATG7 interaction. In addition, ERβ-induced Aβ degradation was blocked by the lysosome inhibitor CQ. Furthermore, DPN, an ERβ agonist, was able to increase cell viability following treatment with Aβ_1–42_ or CQ. Together, our results reveal a new mechanism by which ERβ exhibits its neuroprotection via autophagy activation.

Although several reports have observed that ERβ could regulate autophagy in cancers^24,25^, the effect of ERβ on autophagy in the nerve system is still poorly understood. ERβ, a classical nuclear transcriptional receptor, plays a vital role in the human brain. Savaskan et al.^39^ show an increase in hippocampal ERβ immunoreactivity by immunohistochemistry in AD patients compared with healthy humans. On the contrary, Long et al.^16^ demonstrate that an evident reduction of neuronal ERβ expression was observed in the AD samples than that in normal brains by western blot and immunofluorescence. Our results also shown that ERβ is decreased in AD mice cortex compared with the age-matched wild-type mice (Fig S1A). The contradictory conclusion may be due to the various methods, antibodies, areas of human brains, and stages of patients, suggesting that much research still need to be carried out in the future. Though conflicting evidence exists, the majority of studies indicate that ERβ is a valid therapeutic target for AD treatment. The protective effect of ERβ on AD treatment is mainly regulated by estrogen. However, estrogen deficiency has been regarded as a contributing factor in AD^40^. Based on these data, ERβ activation is considered as a useful strategy for AD therapy. In this study, the human neuroblastoma SH-SY5Y cell line was chosen for the further research, not only because it is widely used as an in vitro model for neuroscience research, but also because it expresses ERβ^41^. We demonstrated that ERβ enhanced the autophagic process in SH-SY5Y cells by increasing autolysosome maturation and autophagic flux, while siRNA of *ERβ* could alleviate EBSS-induced autophagy (Fig. 1). Furthermore, DPN induced-ERβ activation also had a positive effect on autophagy in SH-SY5Y. Taken together, these data suggest that ERβ activates autophagy in SH-SY5Y cells.

A growing body of evidence indicates that autophagy dysfunction contributes to the pathogenesis of AD. Several autophagy-related genes deficiency is shown to enhance the pathology of AD models, such as Beclin1, ATG5, and ATG7^19,42^. Based on the prediction of autophagy regulatory network database and the co-immunoprecipitation experiments, we found ERβ could interact with ATG7 through protein–protein interaction rather than protein–DNA interaction in SH-SY5Y cells (Fig. 3), suggesting a new mechanism for ERβ-mediated autophagy. Given that C/D domain of ERβ [145–255 amino acids] is essential to the protein interaction with Bad Lung Cancer Cells^43^, we explored whether this domain participates in the interaction with ATG7 in SH-SY5Y cells next. As expected, our results showed the C/D domain mainly contributes to ERβ-induced autophagy via ATG7 in SH-SY5Y cells (Fig. 4). Also, considering that C/D domain covers the DNA-binding domain and hinge region of ERβ completely, suggesting a promising drug target for AD treatment. The E1-like activity of ATG7 is necessary to ATG7-regulated autophagy, which coordinates with the E2-like enzyme ATG10 to regulate conjugation of ATG5 to ATG12^34^. Luo et al.^44^ certified that the enzyme activity of ATG7 seems dispensable for the interaction between PSMD10 and ATG7 in hepatocellular carcinoma under stress conditions. However, the present results suggest that ATG7 not only affected the ATG5/ATG12 complex expression but also had a protein–protein interaction with ERβ in SH-SY5Y cells.

Autophagy–lysosome system plays a significant role in the metabolism of Aβ. At present, Aβ degradation enzymes and autophagy are the main Aβ clearance pathways. Multiple studies showed that autophagy facilitates APP degradation and clearance as well as Aβ^45,46^. For example, overexpression of LC3 or Beclin1 reduces both intracellular Aβ accumulation and extracellular Aβ deposition in cellular and the mouse model of AD^19,38^. Moreover, genetic reduction of mammalian target of rapamycin-induced autophagy activation ameliorates the extracellular Aβ deposition in the AD mice model^47^. Our results showed that overexpression of ERβ reduced the extracellular Aβ_1–42_ level in conditioned medium, and this effect was blocked by CQ in SH-SY5Y cells (Fig. 6). It is indicated that impaired lysosomes lost its functions to degrade Aβ_1–42_. Our results indicate that ERβ is important for autophagy-mediated extracellular Aβ degradation. In addition, the ELISA assay showed that the Aβ_1–42_ concentration in cell culture medium of SH-SY5Y was higher than the initial concentration in the presence of CQ, indicating that Aβ degradation is impaired in SH-SY5Y cells. This finding can be explained as follows. Firstly, impaired autophagy stimulates Presenilin-1 expression and enhances γ-secretase activity, leading to Aβ production^48^. On the other hand, CQ breaks the fusion of autophagosome and lysosome, leading to the disruption of lysosomal degradative system and accumulation of AVs. The AVs contain abundantly Aβ, APP, β-secretase, and γ-secretase, suggesting AVs are a key source of Aβ production in AD brains^49^. Moreover, under pathological conditions, Aβ has also been considered as an autophagy inducer through either Akt-dependent pathway or mitochondrial reactive oxygen species generation, resulting in a feedback loop to accelerate Aβ production^50^. These indicate that CQ or transfection reagents bring cytotoxicity to lysosome function, leading to Aβ production and deposition in SH-SY5Y.

In conclusion, we provided evidence of ERβ-mediated autophagy activation via ATG7 is necessary for the degradation of extracellular Aβ_1–42_ in SH-SY5Y cells. In this process, α7nAChR may act as a carrier to bind with extracellular Aβ_1–42_ and then internalize the complex of α7nAChR and extracellular Aβ into cytoplasm, resulting in extracellular Aβ_1–42_ degradation via autophagy in SH-SY5Y. Additional studies are needed to fully understand the role of α7nAChR in ERβ-regulated Aβ clearance via autophagy. Furthermore, results demonstrated that ERβ-induced extracellular Aβ degradation could have neuroprotective roles in SH-SY5Y cells. In summary, our results indicate that ERβ-induced autophagy via ATG7 plays a vital role in extracellular Aβ_1–42_ degradation and defective autophagy may impair Aβ clearance. To our knowledge, these results provide novel insights into the underlying mechanism of the neuroprotective properties of ERβ and generate new mechanisms for the future treatment of AD.

## Supplementary information

supplementary material.

## References

[1] Selkoe DJ (2001). Alzheimer’s disease: genes, proteins, and therapy. Physiol. Rev..

[2] Kuo YM (1996). Water-soluble Abeta (N-40, N-42) oligomers in normal and Alzheimer disease brains. J. Biol. Chem..

[3] Pinho CM, Teixeira PF, Glaser E (2014). Mitochondrial import and degradation of amyloid-beta peptide. Biochim. Biophys. Acta.

[4] Haass C, Selkoe DJ (2007). Soluble protein oligomers in neurodegeneration: lessons from the Alzheimer’s amyloid beta-peptide. Nat. Rev. Mol. Cell Biol..

[5] Mawuenyega KG (2010). Decreased clearance of CNS beta-amyloid in Alzheimer’s disease. Science.

[6] Andersen K (1999). Gender differences in the incidence of AD and vascular dementia: the EURODEM Studies. EURODEM Incidence Research Group. Neurology.

[7] Yue X (2005). Brain estrogen deficiency accelerates Abeta plaque formation in an Alzheimer’s disease animal model. Proc. Natl Acad. Sci. USA.

[8] Carroll JC, Pike CJ (2008). Selective estrogen receptor modulators differentially regulate Alzheimer-like changes in female 3xTg-AD mice. Endocrinology.

[9] Yamaguchi N, Yuri K (2012). Changes in oestrogen receptor-beta mRNA expression in male rat brain with age. J. Neuroendocrinol..

[10] Wilson ME (2002). Age differentially influences estrogen receptor-alpha (ERalpha) and estrogen receptor-beta (ERbeta) gene expression in specific regions of the rat brain. Mech. Ageing Dev..

[11] Wang L, Andersson S, Warner M, Gustafsson JA (2003). Estrogen receptor (ER)beta knockout mice reveal a role for ERbeta in migration of cortical neurons in the developing brain. Proc. Natl Acad. Sci. USA.

[12] Aguirre C, Jayaraman A, Pike C, Baudry M (2010). Progesterone inhibits estrogen-mediated neuroprotection against excitotoxicity by down-regulating estrogen receptor-beta. J. Neurochem..

[13] Yager JD, Davidson NE (2006). Estrogen carcinogenesis in breast cancer. New Engl. J. Med..

[14] Liang K (2010). Estrogen stimulates degradation of beta-amyloid peptide by up-regulating neprilysin. J. Biol. Chem..

[15] Zhao L (2011). 17beta-Estradiol regulates insulin-degrading enzyme expression via an ERbeta/PI3-K pathway in hippocampus: relevance to Alzheimer’s prevention. Neurobiol. Aging.

[16] Long J, He P, Shen Y, Li R (2012). New evidence of mitochondria dysfunction in the female Alzheimer’s disease brain: deficiency of estrogen receptor-beta. J. Alzheimer’s. Dis..

[17] Tanida I (2011). Autophagosome formation and molecular mechanism of autophagy. Antioxid. Redox. Signal..

[18] Guo J (2014). Ginsenoside compound K promotes beta-amyloid peptide clearance in primary astrocytes via autophagy enhancement. Exp. Ther. Med..

[19] Pickford F (2008). The autophagy-related protein beclin 1 shows reduced expression in early Alzheimer disease and regulates amyloid beta accumulation in mice. J. Clin. Investig..

[20] Mueller-Steiner S (2006). Antiamyloidogenic and neuroprotective functions of cathepsin B: implications for Alzheimer’s disease. Neuron.

[21] Wang HC (2015). Autophagy is involved in oral rAAV/Abeta vaccine-induced Abeta clearance in APP/PS1 transgenic mice. Neurosci. Bull..

[22] Li L, Zhang X, Le W (2010). Autophagy dysfunction in Alzheimer’s disease. Neuro-Degener. Dis..

[23] Tarasoff-Conway JM (2015). Clearance systems in the brain-implications for Alzheimer disease. Nat. Rev. Neurol..

[24] Guido C (2012). Estrogen receptor beta (ER beta) produces autophagy and necroptosis in human seminoma cell line through the binding of the Sp1 on the phosphatase and tensin homolog deleted from chromosome 10 (PTEN) promoter gene. Cell Cycle.

[25] Ruddy SC (2014). Preferential estrogen receptor beta ligands reduce Bcl-2 expression in hormone-resistant breast cancer cells to increase autophagy. Mol. Cancer Ther..

[26] Carvalho C, Santos MS, Oliveira CR, Moreira PI (2015). Alzheimer’s disease and type 2 diabetes-related alterations in brain mitochondria, autophagy and synaptic markers. Biochim. Biophys. Acta.

[27] Cai Z, Li B, Li K, Zhao B (2012). Down-regulation of amyloid-beta through AMPK activation by inhibitors of GSK-3beta in SH-SY5Y and SH-SY5Y-AbetaPP695 cells. J. Alzheimer’s. Dis..

[28] Zheng N, Yuan P, Li C, Wu J, Huang J (2015). Luteolin reduces BACE1 expression through NF-κB and through estrogen receptor mediated pathways in HEK293 and SH-SY5Y Cells. J. Alzheimer’s. Dis..

[29] Lioudyno MI (2012). Effect of synthetic abeta peptide oligomers and fluorinated solvents on Kv1.3 channel properties and membrane conductance. PloS ONE.

[30] Kabeya Y (2000). LC3, a mammalian homologue of yeast Apg8p, is localized in autophagosome membranes after processing. EMBO J..

[31] Komatsu M, Ichimura Y (2010). Physiological significance of selective degradation of p62 by autophagy. FEBS Lett..

[32] Skop V (2012). Autophagy–lysosomal pathway is involved in lipid degradation in rat liver. Physiol. Res..

[33] Turei D (2015). Autophagy regulatory network—a systems-level bioinformatics resource for studying the mechanism and regulation of autophagy. Autophagy.

[34] Mizushima N, Sugita H, Yoshimori T, Ohsumi Y (1998). A new protein conjugation system in human. The counterpart of the yeast Apg12p conjugation system essential for autophagy. J. Biol. Chem..

[35] Kim J, Kundu M, Viollet B, Guan KL (2011). AMPK and mTOR regulate autophagy through direct phosphorylation of Ulk1. Nat. Cell Biol..

[36] Jia M, Dahlman-Wright K, Gustafsson JA (2015). Estrogen receptor alpha and beta in health and disease. Best Pract. Res. Clin. Endocrinol. Metab..

[37] Guglielmotto M (2014). Abeta1-42 monomers or oligomers have different effects on autophagy and apoptosis. Autophagy.

[38] Hung SY, Huang WP, Liou HC, Fu WM (2015). LC3 overexpression reduces Abeta neurotoxicity through increasing alpha7nAchR expression and autophagic activity in neurons and mice. Neuropharmacology.

[39] Savaskan E, Olivieri G, Meier F, Ravid R, Muller-Spahn F (2001). Hippocampal estrogen beta-receptor immunoreactivity is increased in Alzheimer’s disease. Brain Res..

[40] Paganini-Hill A, Henderson VW (1994). Estrogen deficiency and risk of Alzheimer’s disease in women. Am. J. Epidemiol..

[41] Bang OY (2004). Neuroprotective effect of genistein against beta amyloid-induced neurotoxicity. Neurobiol. Dis..

[42] Lipinski MM (2010). Genome-wide analysis reveals mechanisms modulating autophagy in normal brain aging and in Alzheimer’s disease. Proc. Natl Acad. Sci. USA.

[43] Zhang G (2010). Ligand-independent antiapoptotic function of estrogen receptor-beta in lung cancer cells. Mol. Endocrinol..

[44] Luo T (2016). PSMD10/gankyrin induces autophagy to promote tumor progression through cytoplasmic interaction with ATG7 and nuclear transactivation of ATG7 expression. Autophagy.

[45] Zhou F, van Laar T, Huang H, Zhang L (2011). APP and APLP1 are degraded through autophagy in response to proteasome inhibition in neuronal cells. Protein Cell.

[46] Son, S. M., Jung, E. S., Shin, H. J., Byun, J. & Mook-Jung, I. Abeta-induced formation of autophagosomes is mediated by RAGE-CaMKKbeta-AMPK signaling. *Neurobiol. Aging* **33**, 1006 e1011–1023 (2012). 10.1016/j.neurobiolaging.2011.09.039 22048125

[47] Caccamo A, De Pinto V, Messina A, Branca C, Oddo S (2014). Genetic reduction of mammalian target of rapamycin ameliorates Alzheimer’s disease-like cognitive and pathological deficits by restoring hippocampal gene expression signature. J. Neurosci..

[48] Ohta K (2010). Autophagy impairment stimulates PS1 expression and gamma-secretase activity. Autophagy.

[49] Yu WH (2004). Autophagic vacuoles are enriched in amyloid precursor protein-secretase activities: implications for beta-amyloid peptide over-production and localization in Alzheimer’s disease. Int. J. Biochem. Cell Biol..

[50] Tung YT (2012). Autophagy: a double-edged sword in Alzheimer’s disease. J. Biosci..

