# Correction: ERβ promotes Aβ degradation via the modulation of autophagy

**DOI:** 10.1038/s41419-019-1867-8

**Published:** 2019-08-23

**Authors:** Yong Wei, Jiawei Zhou, Jun Wu, Jian Huang

**Affiliations:** Hubei Key Laboratory of Cell Homeostasis, College of Life Sciences, Wuhan University, Wuhan, Hubei PR China

**Keywords:** Macroautophagy, Cellular neuroscience

**Correction to: Cell Death & Disease**

10.1038/s41419-019-1786-8 published online 22 July 2019

Following the publication of this article, the authors realized there was an error in Fig. 6h in which two versions of the figure appear. The corrected figure is below. This does not impact the conclusions of the article. We apologize to readers for any confusion this may have caused.

**Fig. 6:**
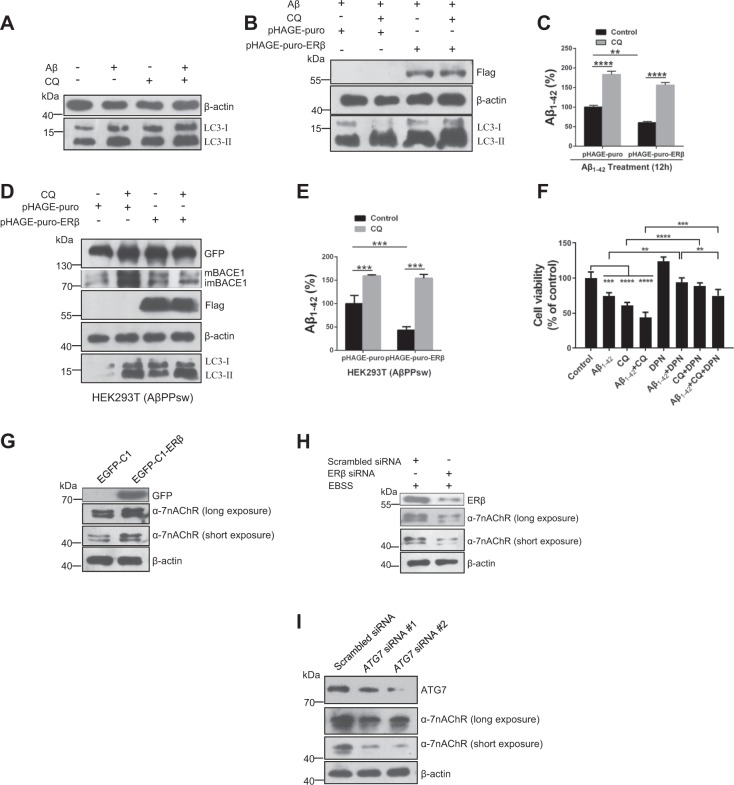
Enhanced autophagy promotes extracellular Aβ_1–42_ degradation in SH-SY5Y cells. **a** Cells were treated with Aβ (1 μM), CQ (10 μM), and Aβ plus CQ for 12 h. Cell lysates were analyzed by immunoblotting for LC3-II and β-actin protein expression. **b** After cells were transiently transfected with pHAGE-puro and pHAGE-puro-ERβ for 24 h, cells were treated with CQ 10 (μM) for 12 h. Next, cells were treated with Aβ_1–42_ Aβ fibrils for 12 h. LC3-II level was tested by western blot. **c** Cells were treated as described in **b**, the Aβ_1–42_ concentration was measured by an ELISA assay. **d** Cells were transfected with ERβ and vector plasmids under CQ treatment or not in the HEK293T (AβPPsw) model. Cell lysates were analyzed by immunoblotting for APP, BACE1, Flag, LC3-II, and β-actin protein expression. **e** Cells were treated as described in **d**, the Aβ_1–42_ concentration was measured by an ELISA assay. **f** Cells were treated with DPN (10 nM) or Aβ_1–42_ (5 μM) for 12 h and then added CQ (10 μM) for another 12 h. The MTT assay was used to test cell viability. **g** Cells were treated as described in (Fig. 1d), then the α7nAChR level was tested by western blot. **h** Cells were treated as described in (Fig. 1b), then the α7nAChR level was tested by western blot. **i** Cells were treated as described in (Fig. S2A), then the α7nAChR level was tested by western blot. Data shown are mean ± S.D. of three independent experiments. (*P < 0.05; **P < 0.01; ***P < 0.001; ****P < 0.0001)

The corrected Fig. 6 and its legend is given below. This has been corrected in both the PDF and HTML versions of the Article.

