# Supplementary material for: ERβ promotes Aβ degradation via the modulation of autophagy
Source: Cell Death Dis. 2019 Jul 22;10(8):565. doi: 10.1038/s41419-019-1786-8 (PMC6646346; doi:10.1038/s41419-019-1786-8)

**ERβ promotes Aβ degradation via the modulation of autophagy**

**Supplementary Figure Legends**

**Figure. S1**

(**A)** ERβ protein expression was detected by western blotting in the cortex lysates from wild-type and 2 x Tg-AD (APPswe/PSENldE9) mice (8 month old, n=6). (**B**) Immunofluorescence assay was used to detect the transfection efficiency of EGFP-C1-ERβ plasmid in SH-SY5Y cells. Scale bar =100 μm. (**C**) Evaluation for LC3-II alteration indicative of autophagy induction in SH-SY5Y cells treated with EBSS for the indicated time. (**D**) Western blot analysis of the LC3-II and SQSTM1 in SH-SY5Y cells treated with various concentrations of DPN (1, 5, 10, 50, 100 nM) for 24 h. Bar graph indicates the relative ratio of LC3-II and SQSTM1 to β-actin in SH-SY5Y cells (right). Data shown are mean ± S.D. of three independent experiments. (*, P<0.05; **, P<0.01; ***, P<0.001).

**Figure. S2**

(**A**) The efficiency of two *ATG7* siRNAs was tested by western blot. (**B**) ATG7 distribution was tested by confocal assay in MEF *Atg7 +/+* and MEF *Atg7 -/-* cells. Scale bar =5 μm. (**C**) ATG7 expression was tested by western blot in MEF *Atg7 +/+* and MEF *Atg7 -/-* cells. (**D**) Cells were treated as described in (**Fig. S1D**), total Ulk1 and phosphorylation of Ulk1 Ser 757 levels were detected by western blot. Data shown are mean ± S.D. of three independent experiments. (*, P<0.05; **, P<0.01; ***, P<0.001).

**Figure. S3**

(**A**) Bar graph indicates the relative ratio of LC3-II to β-actin in **Fig. 6A**. (**B**) Immunofluorescence assay was used to detect the transfection efficiency of EGFP-C1-AβPPsw plasmid in HEK293T cells. Scale bar =100 μm. (**C**) Western blot assay was used to detect APP (GFP tag) and BACE1 protein expression in HEK293T cells. (**D**) ELISA assay was used to measure Aβ_1–42_ concentration in HEK293T (AβPPsw) model. (**E**) Bar graph indicates the relative ratio of α7nAChR to β-actin in **Fig. 6G**. (**F**) Bar graph indicates the relative ratio of α7nAChR and ERβ to β-actin in **Fig. 6H**. (G) Bar graph indicates the relative ratio of α7nAChR and ATG7 to β-actin in **Fig. 6I**. (*, P<0.05; **, P<0.01; ***, P<0.001).

**Figure. S1**


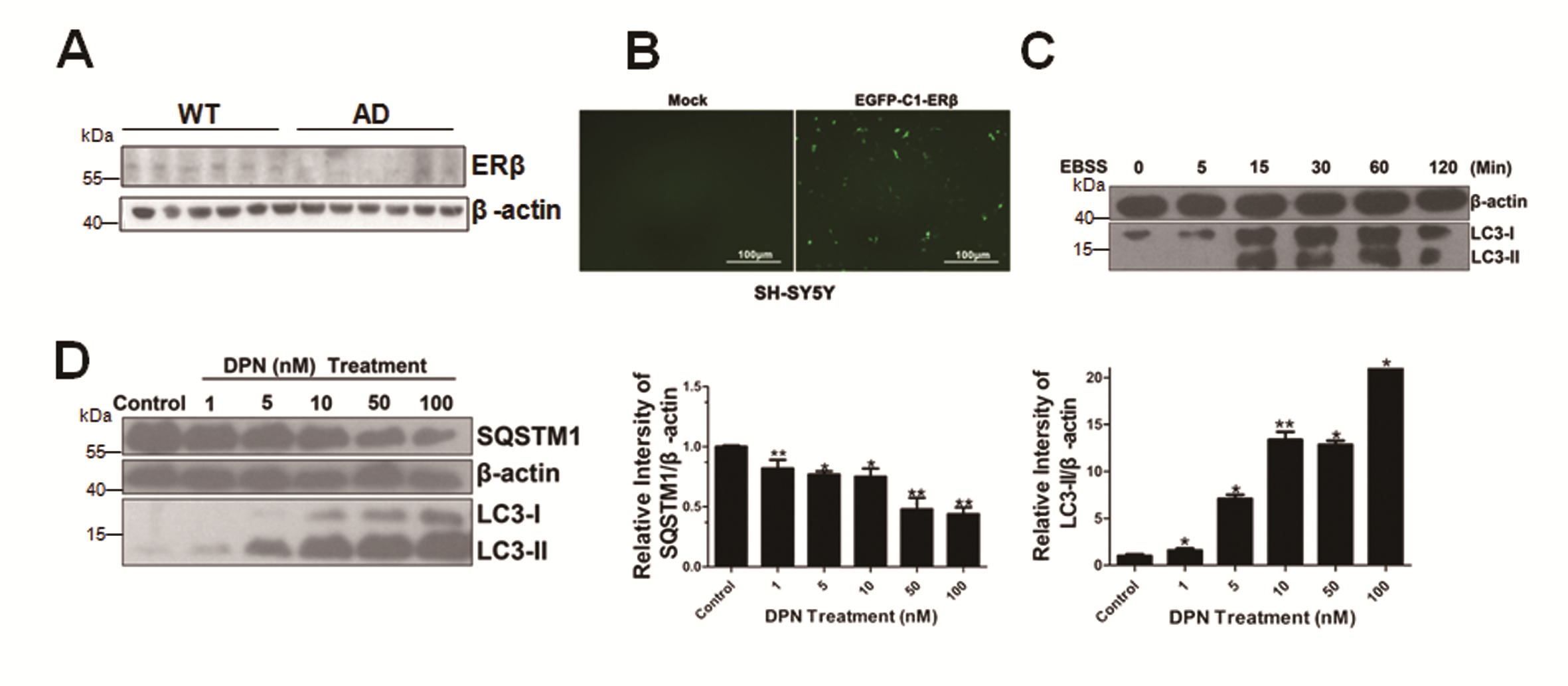


**Figure. S2**


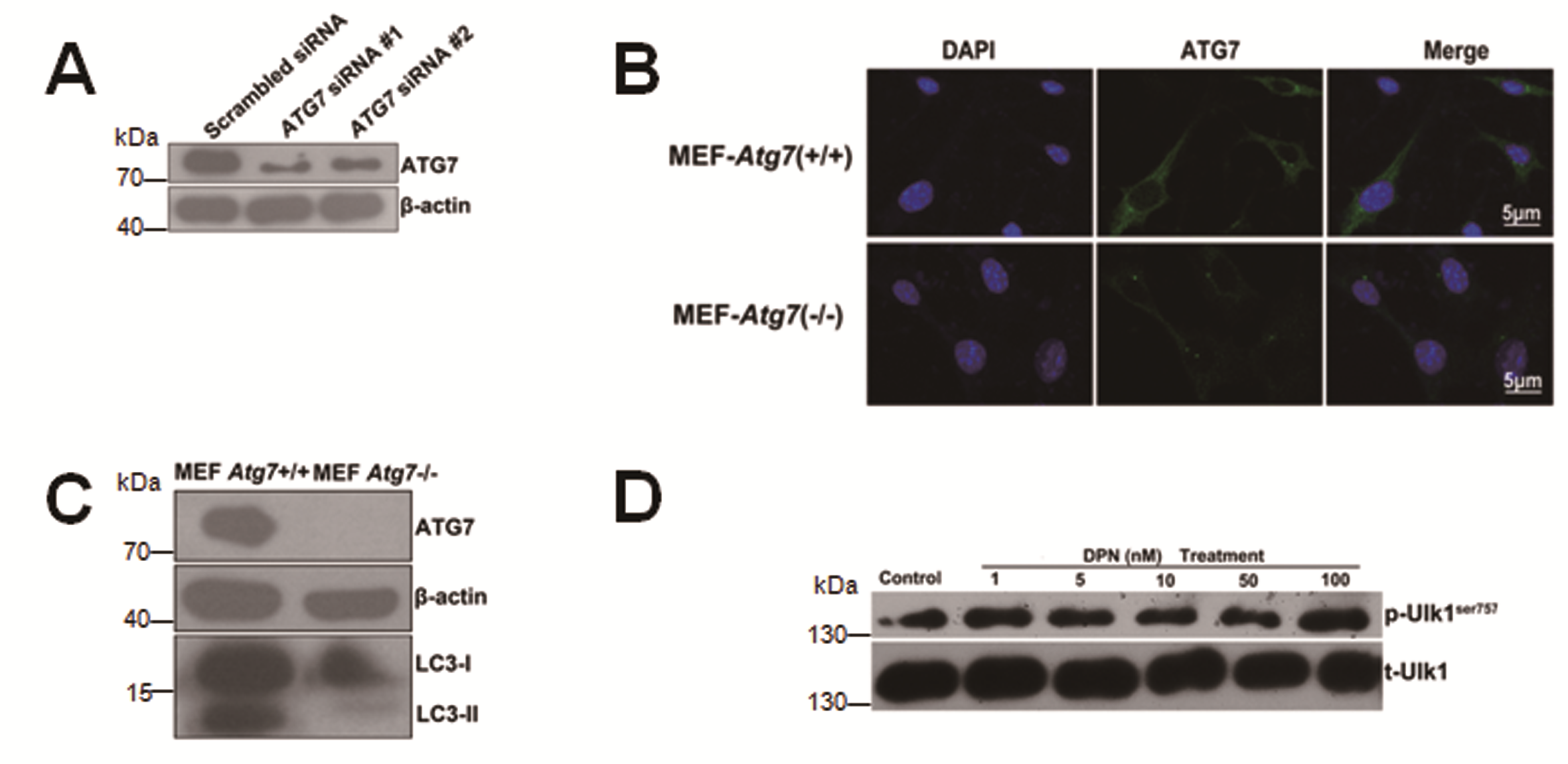


**Figure. S3**


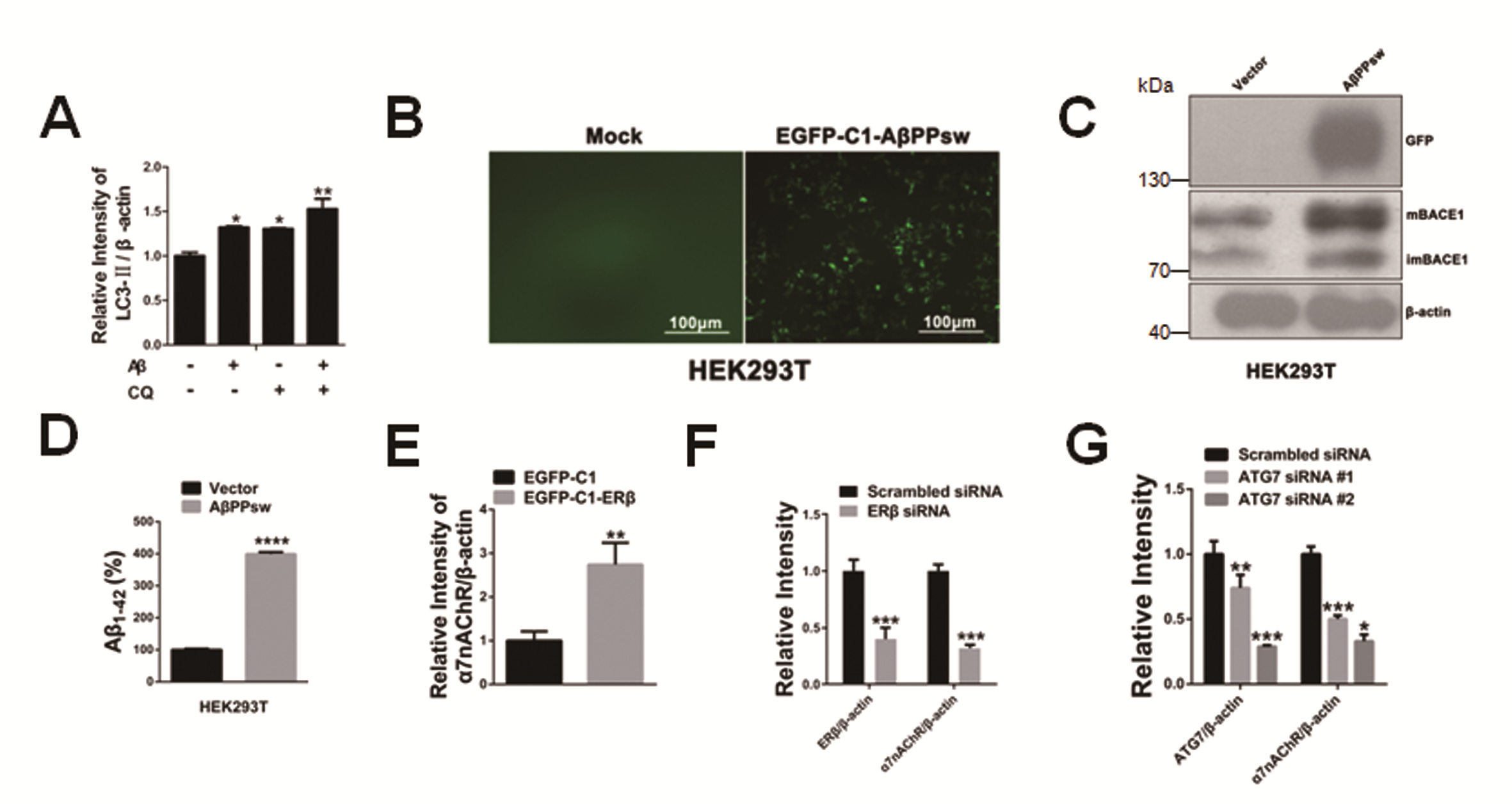

Supplement: Supplementary file 1 — supplementary material. [file 41419_2019_1786_MOESM1_ESM.doc]
